# Numbers of Eurasian beaver (*Castor fiber*) in the Czech Republic, causes of their admission to rehabilitation centres and release rates in the period from 2010 to 2020

**DOI:** 10.1371/journal.pone.0323607

**Published:** 2025-05-15

**Authors:** Gabriela Kadlecová, Filip Kounek, Eva Voslářová, Vladimír Večerek

**Affiliations:** Department of Animal Protection and Welfare and Veterinary Public Health, Faculty of Veterinary Hygiene and Ecology, University of Veterinary Sciences Brno, Brno, Czech Republic; University of Minnesota, UNITED STATES OF AMERICA

## Abstract

The Eurasian beaver (*Castor fiber*) is the largest European rodent. This endangered species is an ecosystem engineer capable of providing several positive impacts in the ecosystems. However, it is also a cause of frequent conflicts with humans. In 2020, the beaver population in the Czech Republic reached 14,610 individuals, with the highest numbers in the Pilsen, Olomouc and Southern Moravian regions. Concurrently, beavers were most often admitted to rehabilitation centres in these regions, and their total numbers in rehabilitation centres increased in the period from 2010 to 2020 (p < 0.01). Beavers were most often admitted after falls into pits and other openings (29.76% of admitted animals) and after a collision with a vehicle (11.9%). Almost half (47.62%) of admitted beavers were released to the wild after their recovery. The mortality rates differed for different causes of admission, with the highest mortality in beavers admitted after a collision with a vehicle (70%) and beavers with bite wounds (67%). There was no significant difference (p > 0.01) in the length of stay in the centres of the beavers that were released to the wild after being treated for the monitored causes of admission. In view of these results, it is important to place particular emphasis on preventive interventions in nature reducing anthropogenic risks for Eurasian beavers and educating the public about the beneficial activities of this endangered species.

## Introduction

After over a hundred years, Europe’s largest rodent, the Eurasian beaver (*Castor fiber*), has returned to Czech landscape. However, the return of this species to the European landscape brings several conflicts with humans, and this problem is often discussed together with the benefits of beaver activity in the landscape [1,2,3,4]. From the point of view of nature protection and biodiversity, the beaver is a crucial species, which is very beneficial in its way of life and activities in the ecosystem. For example, its ability to create extensive wetlands [5] provides suitable conditions for a variety of animal and plant species [6]. Furthermore, it helps to improve the water regime in the landscape, which has long been and is still being disturbed by human activity, especially agriculture [7]. Unfortunately, in today’s changed cultural landscape, the beaver also causes significant damage to water management and agriculture and thus comes into conflict with humans [8,9,10].

Although the Eurasian beaver is listed as a Least Concern species (LC) by the IUCN, according to the Council Directive 92/43/EEC, it is a pan-European protected species requiring strict protection, which requires the declaration of special protection areas. It is also covered by the Bern Convention on the Conservation of European Wildlife and Natural Habitats and indirectly by the Ramsar Convention, which ensures the protection of wetlands of international importance. In the Czech Republic, the legislation classifies it among specially protected animals in the category of highly endangered species [8]. Following the European Union legislation, the Czech Republic adopted the Eurasian Beaver Care Program in 2013. According to this program, the territory of the Czech Republic is divided into three zones (A, B, C), in which different degrees of protection apply. Zone A includes locations of European importance, in which the Eurasian beaver is strictly protected and has the possibility of creating viable populations. These are Český les, Polabí, Litovelské Pomoraví, Chropyňský luh, Niva Dyje, Strážnická Morava and Soutok-Podluží. In areas located in zone B, the key goal is not the protection of beavers, but the connection of populations occurring in zones A. Zone C includes the Vltava basin in South Bohemia (outside the Šumava National Park, where the beaver is protected by law). Given that this is an area with a high number of water management structures, the presence of beavers is undesirable due to the risk of significant damage [11]. The Eurasian Beaver Care Program includes also the concept of compensation for damage according to the Act No. 115/2000 Coll., as amended, on compensation for damage caused by selected specially protected animal species.

From the point of view of the protection of the Eurasian beaver, it is important to educate the public and interest groups, monitor beaver populations and research focused on the influence of the beaver in the landscape in terms of the hydrological regime and interaction in ecosystems [7,8,12]. However, wild beavers are threatened not only by anthropogenic but also by natural factors, such as the effects of weather or infectious diseases, as a result of which beavers are unable to survive in the wild [13,14,15]. Furthermore, beavers were described to suffer from injuries following conflicts between animals of the same species [16], and in some places, predation by other species, such as wolves [17]. Anthropogenic causes of beavers arriving at rehabilitation centres include injuries to beavers on roads [18] and, in the past, hunting [19]. Although Mullineaux and Keeble [20] mention these health problems as common in beavers and describe treatment options and specific health conditions, exact data from rehabilitation centres worldwide on the causes and frequency of beaver admissions remain unknown. Rehabilitation centres care for disabled animals, which are admitted in large numbers every year [21,22,23]. The main goal of these facilities is to release animals after recovery (or in the case of young after rearing) back to nature. The causes of admission are very often closely related to anthropogenic activities [23], which negatively affect the life of wild animals in their natural environment. The operation of rehabilitation centres is especially important in the case of endangered species, which are often among the admitted species [24]. In their case, rescuing each individual and returning it to a suitable habitat can help reinforce unstable populations [25]. In the Czech Republic, rehabilitation centres provide care for disabled animals in all regions. Relevant legislation ensures uniform care at a high-quality level by laying down the conditions for permitting the operation of rehabilitation centres and by the qualification requirements regarding the knowledge and education of operators and workers in these facilities.

The aim of this study was to evaluate the number of Eurasian beavers in the Czech Republic and the trends of their admission to rehabilitation centres, to analyse the reasons for the admission of Eurasian beavers to rehabilitation centres and their outcomes. For the selected causes of admission, the proportion of animals released back into the wild and those who died or had to be euthanized in rehabilitation centres were evaluated. The length of stay according to individual causes of admission and the number of admitted animals in different regions of the Czech Republic were also evaluated. The obtained data and their analysis can better predict the dangers for this species in nature and guide efforts to mitigate their negative impacts. Data on length of stay or mortality rates for some causes of admission may in turn help to improve some procedures in rehabilitation centres with regard to individual welfare.

## Materials and methods

Data on the number of Eurasian beavers in the Czech Republic were obtained from the Nature Conservation Agency of the Czech Republic (NCA). For occurrence data collection, the Nature Conservation Discovery Database (NDOP) system was established and the evaluation protocol https://portal23.nature.cz/publik_syst3/files/monitoring/Mammalia_Postup_2019.pdf was used [26]. The monitoring of beavers was carried out by an active search for locations with existing or new beaver settlements according to monitored residence traces such as dams, lodges, fresh samples of woody plants, tracks, carcasses, or through direct observation according to the methodology of Vorel et al. [27]. Monitoring was carried out in the non-vegetation period, when migration is minimal. The mapping itself was carried out in search of new settlements or confirmation of previously recorded settlements. GPS coordinates were recorded for each finding. Data on Eurasian beavers in the rehabilitation centres were obtained from the central database of the Ministry of the Environment of the Czech Republic. The database contained information on the date of admission of the beavers to the rehabilitation centres and the date when their stay was terminated, the location and the reason of their admission and outcomes. For evaluation purposes, the animals were divided into groups according to the regions where they were found (Pilsen, Olomouc, Southern Moravian, Zlín, Vysočina, Silesia-Moravia, Central Bohemia, Southern Bohemia, Ústí nad Labem, Pardubice, Hradec Králové and Liberec), according to the reason of admission (fall into pits and other openings from which animals cannot escape themselves, traffic collision, bite wounds, exhaustion, rescue transfer, young - dependent on parental care, found in a building, and other - e.g., transfer from another facility) and according to the outcome (release, unassisted death, euthanasia, transfer to another organization, escape and unknown). To evaluate the length of stay, the number of days was calculated as the difference between the date of admission and the date when the stay in the rehabilitation centre was terminated. If it was the same day, the length of stay was set at 0.5 days such as the time required to treat the animal and consider the prognosis. Differences in the length of stay in animals released into the wild for selected reasons for admission were compared (fall into pits and other openings, rescue transfer, bite wounds, exhaustion and found in buildings). The number of released and dead/euthanized Eurasian beavers according to selected causes of admission to rehabilitation centres was also analysed.

The numbers of beavers in the wild and rehabilitation centres in the Czech Republic according to the region where they were found, the reasons for admission and outcomes were compared using the chi-square test with Yates correction in the statistical program UNISTAT 6.5 for Excel (Unistat Ltd., London, UK). As part of the evaluation of the trend in the number of beavers admitted to the rehabilitation centres during the observed period and the comparison of the number of beavers in the wild and the number of admissions to the rehabilitation centres between regions, a Spearman correlation test was performed. Differences in the length of stay were compared by Kruskal-Walis ANOVA. A value of p < 0.05 was determined to be statistically significant.

## Results

**Fig 1:**
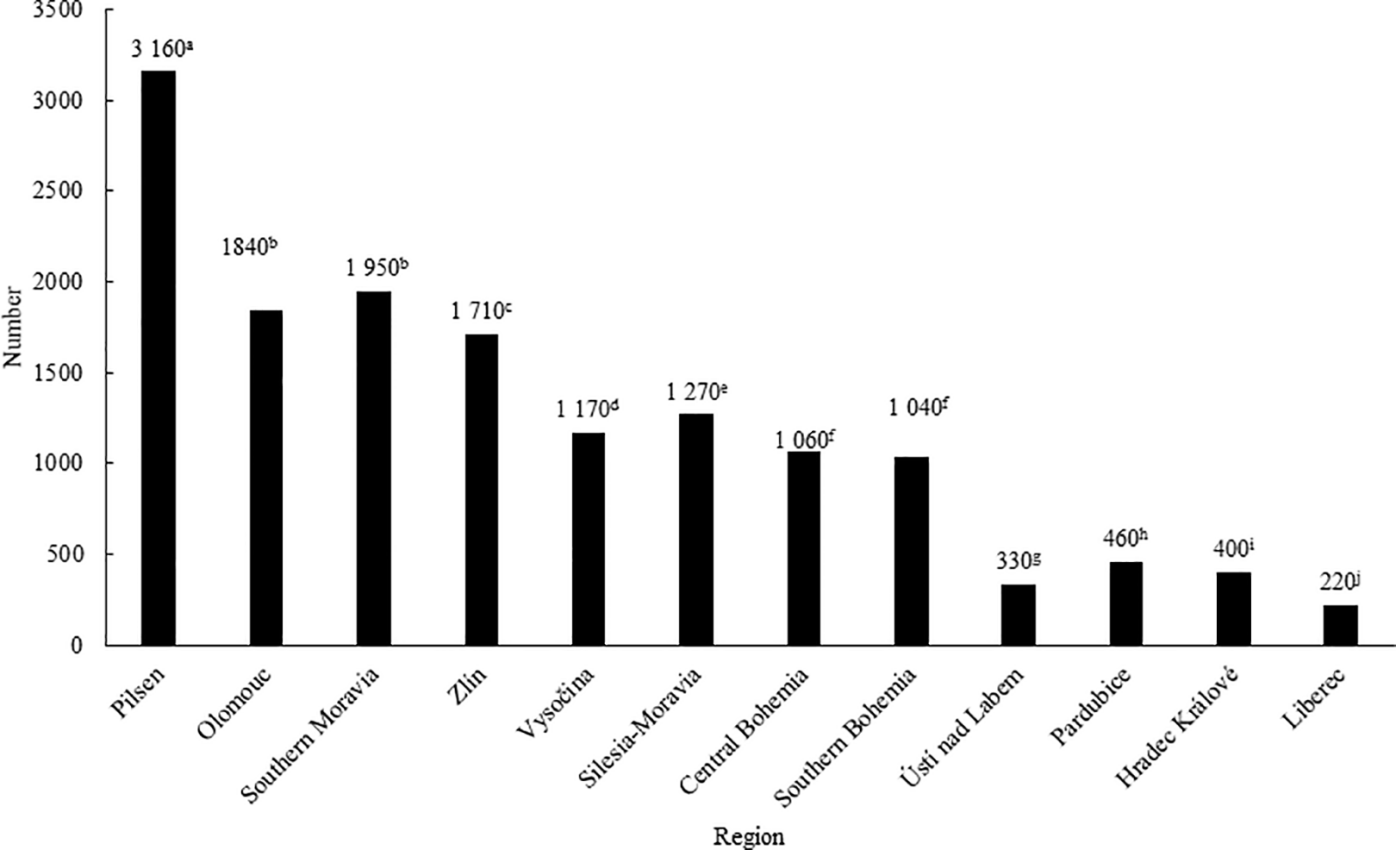
Number of Eurasian beavers in individual regions of the Czech Republic in 2020. ^a-j^The values with different superscript letters are significantly (p < 0.05) different.

In 2020, 14,610 Eurasian beavers were recorded on the territory of the Czech Republic (Fig 1), with the largest (p < 0.05) population found in the Pilsen Region (3,160 animals), followed by the Southern Moravian Region (1,950 animals) and Olomouc Region (1,840 animals). On the other hand, the smallest population (p < 0.05) when compared to the other regions was found in the Ústí nad Labem Region (330 animals), Pardubice Region (460 animals), Hradec Králové Region (400 animals) and Liberec Region (220 animals).

**Fig 2:**
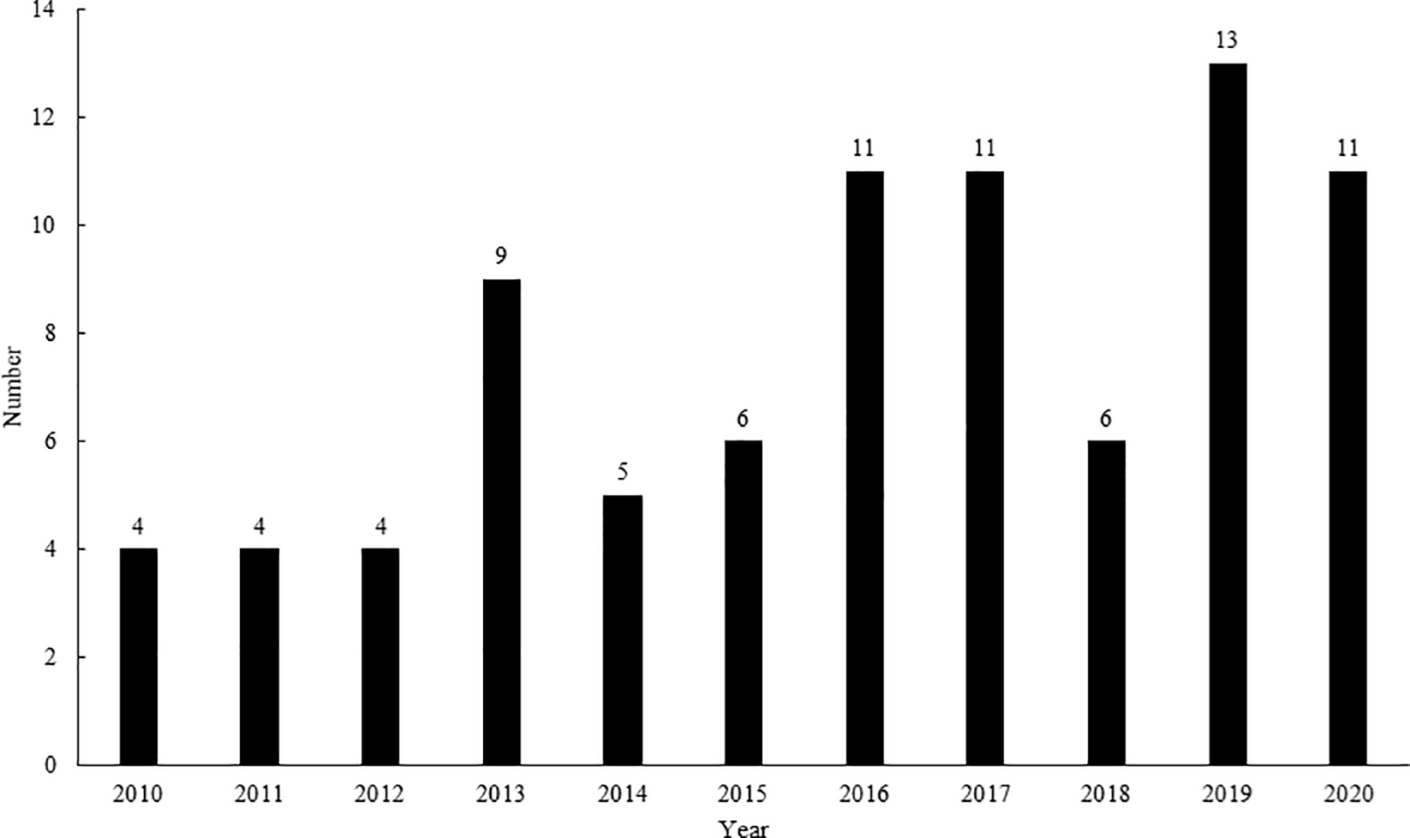
The number of Eurasian beavers admitted to rehabilitation centres in the Czech Republic in the period from 2010 to 2020.

**Fig 3:**
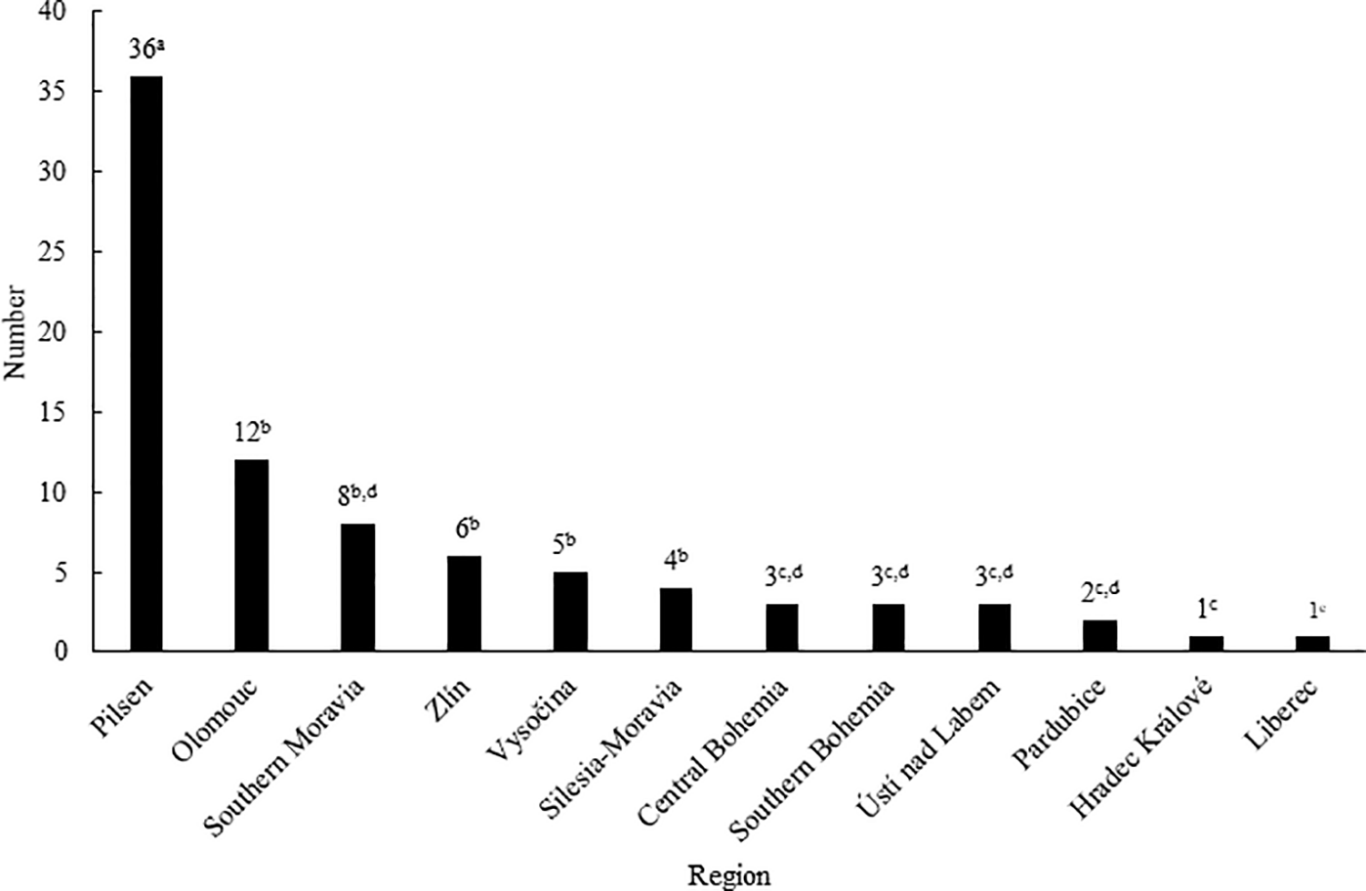
Number of Eurasian beavers admitted to rehabilitation centres in the Czech Republic by individual region in the period 2010-2020. ^a-j^The values with different superscript letters are significantly (p < 0.05) different.

A total of 84 Eurasian beavers were admitted to 21 rehabilitation centres in the Czech Republic during the period 2010–2020 (Fig 2). An increasing trend (rSp = 0.8401, p < 0.05) in the number of animals admitted was found during the monitored period. Until 2015, admissions of beavers were rare, except in 2013 when 9 animals were admitted. However, from 2016 more than 10 animals were admitted every year, except in 2018 when only 6 beavers were admitted. The numbers of admitted beavers differed in individual regions (Fig 3). The highest number (p < 0.05) of beavers were admitted in the Pilsen region. A positive correlation was found between the number of Eurasian beavers admitted to rehabilitation centres and the number of beavers recorded in the wild in individual regions (rSp = 0.9383, p < 0.05).

Beavers were admitted to rehabilitation centres most frequently (p < 0.05) after falling into pits and other openings (Table 1).

**Table 1:** The reason for the admission of Eurasian beavers to rehabilitation centres in the Czech Republic in 2010-2020.

Reason for admission	Number	%
Falls into pits and other openings	25	29,76^a^
Traffic collision	10	11,9^b^
Bite wounds	6	7,14^b^
Exhaustion	5	5,95^b^
Rescue transfer	5	5,95^b^
Young	4	4,76^b^
Found in a building	3	3,57^b^
Other	26	30,95^a^

a,bThe values with different superscript letters in a column are significantly (p < 0.05) different.

Outcomes for Eurasian beavers in the rehabilitation centres in the Czech Republic are shown in Table 2. The highest number of beavers (p < 0.05) were released back into the wild (47.62%). A relatively high percentage of admitted beavers (25%) died, while only a small proportion was euthanized (1.19%).

**Table 2:** Outcomes for Eurasian beavers in the rehabilitation centres in the Czech Republic in the period 2010-2020.

Outcome	Number	%
Release	40	47,62^a^
Unassisted death	21	25,00^b^
Transfer to another organisation	2	2,38^c^
Escape	2	2,38^c^
Euthanized	1	1,19^c^
Unknown	16	21,43^b^

a-cThe values with different superscript letters in a column are significantly (p < 0.05) different.

There were differences in outcomes for beavers admitted for monitored reasons (Table 3). Significantly more (p < 0.05) beavers found in pits and other openings were subsequently released into the wild (84%) in comparison with release rates of beavers admitted due to exhaustion (60%), traffic collision (20%) and bite wounds (17%). While all beavers found in a building were released (100%), no beavers admitted as young were released during the monitored period (0%). The mortality was highest in beavers admitted after traffic collision (70%) and due to exhaustion (40%).

**Table 3:** Numbers of released and dead or euthanized Eurasian beavers according to selected causes of admission to rehabilitation centres in the Czech Republic in 2010-2020.

Reason for admission	Admitted in total (number)	Release		Death/euthanasia	
		Number	%	Number	%
Falls into pits and other openings	25	21	84^a^	2	8^x^
Traffic collision	10	2	20^b^	7	70^x,y^
Bite wounds	6	1	17^b^	4	67^y^
Exhaustion	5	3	60^b^	2	40^x,y^
Young	4	0	0^b^	1	25^y^
Found in a building	3	3	100^b^	0	0^x,y^

a, b*The values with different superscript letters in a column are significantly (p < 0.05) different.*

x, y*The values with different superscript letters in a column are significantly (p < 0.05) different.*

**Fig 4:**
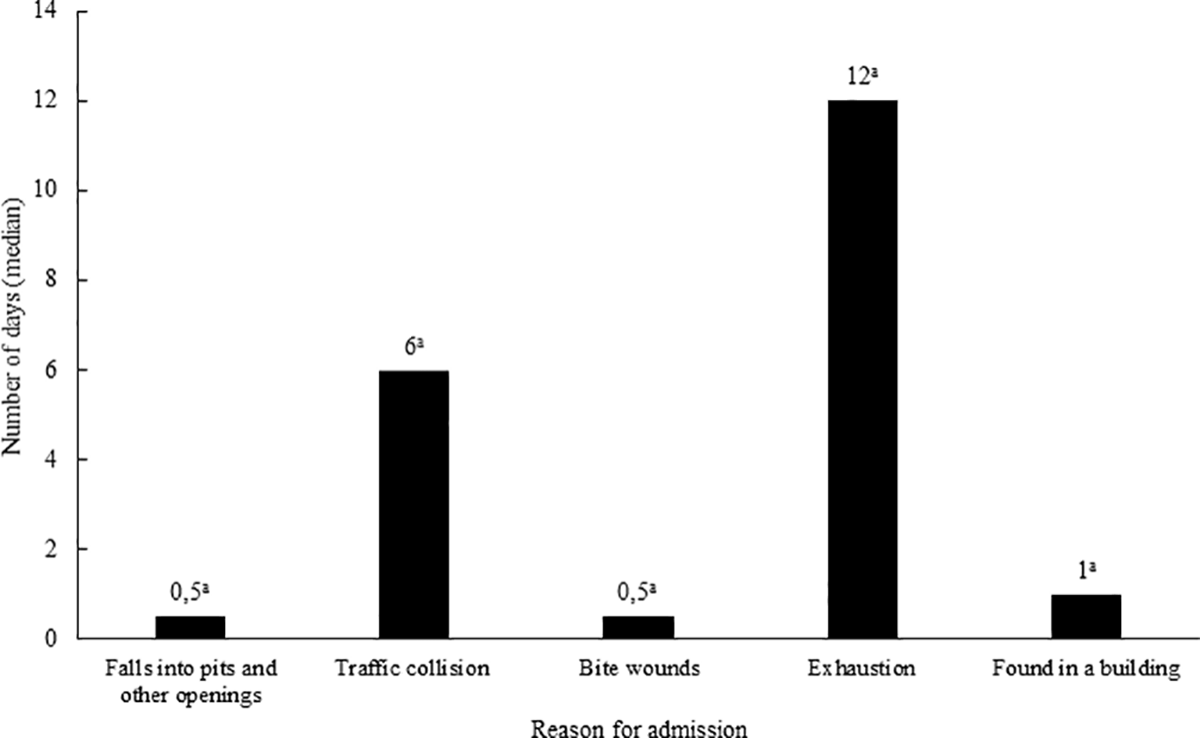
Length of stay by reason of admission of Eurasian beavers that were subsequently released into the wild from rehabilitation centres in the Czech Republic in the period 2010-2020. ^a^The values with the same superscript letters in are not significantly (p > 0,05) different.

The median length of stay of beavers in the rehabilitation centres until they were released was 1 day. No significant (p > 0.05) difference was found in the length of stay of beavers admitted for different reasons (Fig 4).

## Discussion

In 2020, the Eurasian beaver population in the Czech Republic reached 14,610 individuals, which may be a sign of the positive impact of the protection of this species in the given area [28], as the estimated number in 2012 was only around 3,000 [29]. In the 18th century, the beaver was almost hunted to extinction in European territory for its fur, meat, and castoreum used in perfumery and medicine. Another reason for a decrease in their population was a direct conflict with humans due to damage caused especially in pond farming [11]. Reintroduction efforts were made in the 19th century, but were unsuccessful, and by the end of the 19th century, only 1,200 beavers remained in their original European range [30]. Nevertheless, for example, Nolet and Rossel [30] state that the viable population of European beavers is at least 1800 individuals in the population. In contrast, in Europe in 1997, there were around 430,000 beavers [30] and in 2012 their population reached one million [19]. In 2020 in the Czech Republic, the highest numbers of beavers were recorded in the Pilsen, Olomouc and Southern Moravian regions, which is probably related to the increased protection of this species in these areas. In these regions, most of the Europeanly important areas belonging to the so-called zone A are located, i.e., the Eurasian beaver is strictly protected there and can form continuous viable populations. Another reason for the higher numbers in these regions is the way of settlement of the Czech territory, which began in the 1970s with the migration of beavers from Bavaria and Saxony, Austria and the reintroduction of beavers in the Olomouc region in the Litovelské Pomoraví Protected Landscape Area. Records of the occurrence of the Eurasian beaver in the Czech Republic in 2000 show that at that time this species mainly inhabited the above-mentioned regions and from there it gradually spread over almost the entire territory of the Czech Republic [29]. Reintroducing beavers to their former habitats, where populations were present in the past, is a subject of much consideration, including deciding on the proper release location and decisions at the national level, along with intensive follow-up monitoring and public education about the positive impacts of this species on nature [31].

Within the monitored eleven-year period, an increasing trend in the number of beavers admitted to rehabilitation centres in the Czech Republic was found, which coincides with trends reported for other species in the Czech Republic [24,32] as well as the trends seen in rehabilitation centres in other countries [22]. A positive correlation (p < 0.05) was found between the number of beavers recorded in the wild in individual Czech regions and the number of beavers admitted to rehabilitation centres, i.e., in the regions with the largest wild beaver population, the highest number of beavers admitted to rehabilitation centres was recorded. However, it might be not only a consequence of the higher occurrence of beavers in the given area but also the fact that in these European important areas, along with the stricter protection, the surveillance of the endangered species is also strengthened, for example in the form of regular checks of risky places where beavers can get stuck. The increasing numbers of admitted beavers over the entire eleven-year period may result from increasing risks affecting the survival and health of animals in the wild compared to the past, and thus also the probability of acquiring disability leading to an admission to rehabilitation centres. In the wild, animals are increasingly affected especially by factors associated with anthropogenic activity [33] which are also frequent causes of admission to rehabilitation centres, an example can be the increasing mortality on roads due to the intensive construction of roads [34,35,36]. It can also be a greater effort to save disabled animals, especially in the case of endangered species, for which awareness is spreading among the lay public as well. Although the beaver has a reputation for many people as an unwelcome pest and an unnecessary and undesirable species in today’s cultural European landscape, its positive impact on the landscape is not negligible [37]. The ability to create wetlands and water bodies around watercourses significantly helps to retain water in the landscape and positively affects the hydrological balance [38]. Biotopes created by beaver activity help maintain or increase the biodiversity of wetland ecosystems and the species of animals and plants associated with them and can often significantly enrich anthropocenoses [37]. Beaver dams on watercourses can significantly affect the rise of groundwater [39], slow down the flow rate, create meanders and retain water in the landscape, which can lead to a change in the composition of the vegetation and a positive effect on the natural landscape including partial flood protection [40]. However, in the agricultural landscape, it has a negative impact on farmed land [41], which cannot be used after flooding. Surveys have shown that streams with beaver dam systems have much richer invertebrate communities [6,8]. Newly created pools and water bodies become essential for amphibians [42], who find suitable places for breeding and hunting here. There is a constant succession and change in the sites transformed by the beaver, which is a very important factor for amphibians to occupy a suitable habitat and increase their species diversity [38]. Beaver habitats are also positive for a number of aquatic bird species [43,44,45] - for Gruiformes, Anseriformes, Piciformes, Charadriiformes, which look for insects in dead tree trunks. Last but not least, these habitats attract small mammals, for which they represent attractive places with a rich food resource. As a result of the food requirements of the beaver, the composition of the riparian vegetation also changes, and in some places, light-loving species of plants may prevail over time, which can provide a year-round source of food for some large ungulates [37]. According to Hartman [46], large herbivores can act as food competitors to beavers by overgrazing softwoods and grazing their regenerating shoots thus eliminating the development of beaver populations in a certain area. In the Czech Republic, for the time being, only bites from roe deer or deer on felled trees have been recorded at beaver sites [8]. Rehabilition centres seek to educate the public about these facts through various communication channels to foster a more positive perception of beavers in the Czech Republic. However, beavers are often viewed negatively by farmers. Efforts are being made to mitigate their concerns, primarily through compensation for damage caused by the species, as stipulated in Czech legislation for other specially protected animals. Collaboration between conservationists, land users (such as farmers), and the general public is crucial in this regard [47]. However, the direct damage caused by beavers´ feeding is relatively minor [48]. They primarily graze in the summer, and vegetation can constitute a significant portion of their diet [49].

Most beavers were admitted to the rehabilitation centres after being found in pits or other openings, namely 29.76% of all beavers admitted during the monitored period. The cause of falls can usually be artificial holes dug in the ground, an example is melioration in fields that the animal cannot see and has no chance to get out of by itself. In general, habitat fragmentation and migration barriers reduce the permeability of the landscape and force beavers to migrate overland away from watercourses, which greatly increases the risk of injury, death or entanglement in man-made objects. The collisions with traffic were the second most frequent cause of admission (11.90% of admitted beavers). Collisions with vehicles are reported in many species as one of the most serious reasons for their mortality in the wild [34], which can vary depending on time and demographic distribution of the landscape [50,51]. The construction of roads and highways also significantly fragments the landscape and thus creates more opportunities for collisions with animals, especially if they are territorial animals [52]. For beavers, the highest risk is during spring migrations, when especially young animals (aged 1–3 years) are looking for new territories, which can be very demanding [37,53]. If they fail to find a new refuge near their native territory, they can travel up to tens of kilometres even outside the aquatic environment and overcome different altitudes [54,55] before they are successful, often with aggressive fights for the new territory. Considerably exhausted and, in some cases, injured beavers more often become victims of traffic accidents, or are found in non-traditional places (in buildings, cesspools, reservoirs) [37]. In the Czech Republic, the growing beaver population may also be leading to a gradual expansion of their territories closer to roads and railway lines [56]. Road mortality has a significant impact on animal populations, and in beavers, it was determined to be the most common cause of death in Germany in the 1930s [18]. Collision with a vehicle is usually fatal for small and medium-sized mammals, however, the animal can sometimes survive and suffer injuries, especially to the peripheral parts of the body [57].

A relatively large number of Eurasian beavers (47.62%) were successfully cured and released back into the wild, i.e., the main goal of the rehabilitation centres was fulfilled. The highest release rate was found for beavers found in pits and other openings, which was also the most common reason for admission. Therefore, it seems that the fall itself does not usually cause serious injuries or those that would be difficult to treat and would end in death or euthanasia, as the finding of a beaver in a pit led to death or euthanasia in only 2 cases out of 25. When animals are trapped in places where they cannot get out, it is especially important to find them in time, to prevent exhaustion and starvation, which can lead to a worse response of the organism to subsequent treatment and a stay in the rehabilitation centres. In connection with the construction of land reclamations, canals and other traps for animals, it is especially important to regularly check them, to secure the site as a prevention against the entry and trapping of wild animals and also to educate the general public, as such traps can also be found in gardens and other private spaces (for example, when digging a well). Although short-term starvation may not critically affect beavers [58], prolonged entrapment, particularly in extreme temperatures, can be dangerous - especially when these areas are not monitored, causing the animals to remain trapped for extended periods. Rehabilitation centres in the Czech Republic also managed to release every beaver that was found in a building. Such cases usually do not involve disabled animals, however, an intervention of a competent person is necessary to safely capture the beaver, as beavers when cornered can be aggressive. In addition, it is an endangered species in the Czech Republic, and thus, any handling of beavers must be documented. There is also generally more care given to the choice of a place where an animal of endangered species is released in order to ensure that it is as optimal as possible to preserve the life and health of the animal and its integration into existing populations, which are usually at risk. Out of exhausted beavers, i.e., those admitted due to long-term deprivation and starvation, 60% were released. Ideally, the health of beavers should be monitored and assessed after their release. However, this requires either recapturing the animals [59] or at least determining the causes of mortality to rule out improper release procedures, such as releasing individuals that were not yet healthy or mature enough.

Mortality rates of beavers in the rehabilitation centres resulted mainly from their unassisted death (25%), only one animal was euthanized. The results indicate a great effort of rehabilitation centre workers in cooperation with veterinarians to save every beaver given it is an endangered species in the Czech Republic. Concerning treatment vs. euthanasia decisions, however, it is always important to estimate prognosis. Especially in wild animals, any handling by humans, necessary for treatment, is a severe stressor that significantly affects their welfare. Stress, particularly chronic stress, is known to negatively affect the immune system of animals [60]. The presence of other animals, including predator species, as well as the frequent presence of caregivers, also contribute to chronic stress in rehabilitation centres. Although no studies have specifically examined stress levels in beavers at rehabilitation centres – such as the effects of injury type or the presence of other animals near exclosures – stress induced behavioral changes can negatively impact treatment. For example, stress may lead to food refusal, making oral administration of medications difficult and increasing the need for handling during drug administration. However, frequent handling is undesirable, and beavers should be handled as little as possible. Therefore, providing a calm and suitable environment is crucial for their well-being [20]. A high infection pressure in these facilities associated with a high concentration of diseased animals can also have a negative effect. Early euthanasia of animals with a little chance for recovery will prevent them from prolonged suffering. The highest mortality was found in beavers admitted after collisions with a vehicle (70%) and due to bite wounds (67%). Injuries caused by another animal may result from fights among beavers, as they are strictly territorial and do not hesitate to attack any intruder in their territory [33]. Their teeth, adapted for gnawing trunks, can cause serious injuries and they easily become infected [16], causing complications and treatment difficulties in the healing of these wounds. Bite wounds can be also caused by their natural predators such as wolf (*Canis lupus*), bear (*Ursus arctos*) and lynx (*Lynx lynx*) [61,62]. Separation from other beavers with whom the individual lived also has a negative impact on the welfare of captured beavers treated in rehabilitation centres [63]. Beavers are not only susceptible to infections from open wounds but can also host a wide range of pathogens, including those with zoonotic potential [64,65,66]. Therefore, rehabilitation center workers should avoid unprofessional interventions that may increase the risk of disease transmission. In the Czech Republic, these workers are required to be knowledgeable not only about the protection and welfare of injured animals but also about safeguarding their own health and that of their colleagues, with regular training mandated. Additionally, beavers can be aggressive when handled [20], making it essential to wear protective gloves and use appropriate handling tools. A high mortality rate among beavers in Czech rehabilitation centres aligns with findings by [18], who reported mortality in wild beavers due to vehicle collisions, entrapment, and, among non-anthropogenic causes, infectious diseases.

The length of stay of animals in rehabilitation centres may related to the reasons for which they were admitted there [22]. In our study, no difference (p > 0.05) was found between the median length of stay of beavers admitted for different reasons. The beavers admitted due to falls into pits and bite wounds stayed in the rehabilitation centres for 0.5 day (median) before they were released. The beavers found in a building spent in the rehabilitation centres 1 day (median) before they were released. A high proportion (84%) of beavers released after being found fallen in pits and their short length of stay in the rehabilitation centres confirm an assumption that when falling beavers usually suffer only minor injuries. For beavers trapped in pits is also important to be found in time. Farmers or other persons who are aware of the presence of beavers in the area may check the places presenting a risk to animals more frequently or trapped animals may be found just because they got trapped in places frequently visited by humans. When beavers are not found in time, they die without being discovered. Nevertheless, the chances of beavers returning to the wild when found in the pit are high, which is very encouraging considering the largest number of beavers was admitted for this reason. The beavers admitted exhausted spent 12 days (median) in the rehabilitation centres before they could be released. The care provided in the rehabilitation centres resulted in 60% of beavers recovering and being released. The median stay until the release of beavers admitted after a collision with a vehicle was 6 days according to Fig 4. This figure suggests that beavers that were released suffered only minor injuries that could be resolved in a relatively short time, as opposed to more serious injuries that appeared to be mostly fatal regardless of the treatment efforts, as indicated by the number of animals that died.

The goal of the rehabilitation centres and other organizations supporting the survival of endangered species in nature should be creating conditions under which even species involved in human-wildlife conflict, such as beaver, may thrive [67]. Safeguarding beavers goes beyond preserving the species; it also plays a crucial role in protecting wetlands. These wetlands are vital components of nature and serve as one of the most important ecosystems, supporting a diverse range of organisms globally. The return of beavers to the Czech and Central European landscapes doesn’t merely signify potential damage to agriculture and water management. More importantly, it heralds the return of a species capable of positively transforming the landscape and enhancing biodiversity.The return of beavers to the Czech and Central European nature does not only mean damage to agriculture and water management but above all the return of species that can positively transform the landscape and increase biodiversity.

## Conclusion

The results document that most beavers are admitted to rehabilitation centres located in areas with the highest beaver population. A higher number of animals means also a higher movement of animals in the area and an increased risk of collisions with traffic, injuries resulting from fights over the territory or falling into pits and other openings. Traffic collisions and falls into pits were found to be the most frequent causes of admission of beavers to rehabilitation centres and also the most frequent causes of their death.

The population of beavers in the Czech Republic is increasing, despite this, it is still an endangered species, just like in many other countries, in which case every saved individual can be of great importance for strengthening the population. For this reason, it is necessary to focus on risk factors related to their admission to rehabilitation centres or the provision of wildlife corridors by the roads, as well as the minimization of disturbing factors and conflicts with human activity that force these animals to migrate more often or fight for territories.

## References

[1] Brazier RE, Puttock A, Graham HA, Auster RE, Davies KH, Brown CML. Beaver: nature’s ecosystem engineers. WIREs Water. 2021;8(1):e1494. doi: 10.1002/wat2.1494 33614026 PMC7883483

[2] Hohm M, Moesch S, Bahm J, Haase D, Jeschke J, Balkenhol N. Reintroduced, but not accepted: stakeholder perceptions of beavers in Germany. People Nat. 2024;6:1681–95.

[3] Rosell F, Bozsér O, Collen P, Parker H. Ecological impact of beavers *Castor fiber* and *Castor canadensis* and their ability to modify ecosystems. Mammal Rev. 2005;35:248–76.

[4] Oliveira S, Buckley P, Consorte-McCrea A. A glimpse of the long view: human attitudes to an established population of Eurasian beaver (*Castor fiber*) in the lowlands of south-east England. Front Conserv Sci. 2023;3:925594.

[5] Macfarlane WW, Wheaton JM, Bouwes N, Jensen ML, Gilbert JT, Hough-Snee N, et al. Modeling the capacity of riverscapes to support beaver dams. Geomorphology. 2017;277:72–99. doi: 10.1016/j.geomorph.2015.11.019

[6] Bartel RA, Haddad NM, Wright JP. Ecosystem engineers maintain a rare species of butterfly and increase plant diversity. Oikos. 2010;119(5):883–90. doi: 10.1111/j.1600-0706.2009.18080.x

[7] Viviano A, Mazza G, Di Lorenzo T, Mori E. Housed in a lodge: occurrence of animal species within Eurasian beaver constructions in central Italy. Eur J Wildl Res. 2022;68:75.

[8] Vorel A, Šíma J, Uhlíková J, Peltánová A, Mináriková T, Švanyga J. Program péče o bobra evropského v České republice. Praha: AOPK ČR a MŽP ČR; 2013.

[9] Trentanovi G, Viviano A, Mazza G, Busignani L, Magherini E, Giovannelli A, et al. Riparian forests throwback at the Eurasian beaver era: a woody vegetation assessment for Mediterranean regions. Biodivers Conserv. 2023;32:4259–74.

[10] Dvořák J. Diet preference of Eurasian Beaver (*Castor Fiber* L., 1758) in the environment of Oderské vrchy and its influence on the tree species composition of river bank stands. Acta Univ Agric Silvic Mendelianae Brun. 2013;61(6):1637–43. doi: 10.11118/actaun201361061637

[11] Svobodová J. Bobr evropský v České republice. Myslivost. 2023;4:68.

[12] Auster R, Puttock A, Brazier R. Unravelling perceptions of Eurasian beaver reintroduction in Great Britain. Area. 2020;52:364–75.

[13] Turillazzi F, Mori E, Viviano A, Baratti M, Pucci C, Gobbi M. Beavers are not alone: parasitic assessment of released Eurasian beavers in central Italy. Mamm Res. 2024;69:33–41.

[14] Åhlen P-A, Sjöberg G, Stéen M. Parasitic fauna of Eurasian beavers (Castor fiber) in Sweden (1997-1998). Acta Vet Scand. 2021;63(1):23. doi: 10.1186/s13028-021-00588-w 34078419 PMC8176557

[15] Bystrianska J, Papajová I, Šmiga Ľ, Šoltys J, Majláthová V, Majláth I. First report on parasites of European beavers in the Slovak Republic. Parasitol Res. 2021;120:355–8. 33128643 10.1007/s00436-020-06943-6

[16] O’Brien MF, Meldrum J, Foster I. Medical and surgical management of intraspecific wounds in a European beaver kit (*Castor fiber*). Vet Record Case Reports. 2018;6(1). doi: 10.1136/vetreccr-2017-000561

[17] Gable TD, Windels SK, Romanski MC, Rosell F. The forgotten prey of an iconic predator: a review of interactions between grey wolves *Canis lupus* and beavers *Castor* spp. Mammal Rev. 2018;48(2):123–38. doi: 10.1111/mam.12118

[18] Stefen C. Causes of death of beavers (Castor fiber) from eastern Germany and observations on parasites, skeletal diseases and tooth anomalies—a long-term analysis. Mamm Res. 2019;64:279–88.

[19] Halley DJ, Rosell F. The beaver’s reconquest of Eurasia: status, population development and management of a conservation success. Mammal Rev. 2002;32(3):153–78. doi: 10.1046/j.1365-2907.2002.00106.x

[20] Mullineaux E, Keeble EJ. BSAVA manual of wildlife casualties. 2nd edition. Quedgeley: British Small Animal Veterinary Association; 2016.

[21] Garcês A, Soeiro V, Lóio S, Sargo R, Sousa L, Silva F. Outcomes, mortality causes, and pathological findings in European hedgehogs (*Erinaceus europeus*, Linnaeus 1758): a seventeen year retrospective analysis in the north of Portugal. Animals. 2020;10:1305. 32751458 10.3390/ani10081305 PMC7460247

[22] Molina-López RA, Mañosa S, Torres-Riera A, Pomarol M, Darwich L. Morbidity, outcomes and cost-benefit analysis of wildlife rehabilitation in Catalonia (Spain). Margalida A, editor. PLoS One. 2017;12(7):e0181331. doi: 10.1371/journal.pone.0181331 28719647 PMC5515437

[23] Schenk AN, Souza MJ. Major anthropogenic causes for and outcomes of wild animal presentation to a wildlife clinic in East Tennessee, USA, 2000-2011. PLoS One. 2014;9(3):e93517. doi: 10.1371/journal.pone.0093517 24686490 PMC3970955

[24] Lukesova G, Voslarova E, Vecerek V. Mammals at rescue centres in the Czech Republic: trends in intake and outcome, causes of admission, length of stay and release rate. J Nat Conserv. 2022;67:126156.

[25] Tracy L, Wallis G, Efford M, Jamieson I. Preserving genetic diversity in threatened species reintroductions: how many individuals should be released? Anim Conserv. 2011;14:439–46.

[26] Bobr evropksý. In: AOPK, ISOP Fcited Czech Republic. [10 March 2025]. Available from: https://portal23.nature.cz/publik_syst3/files/monitoring/Mammalia_Postup_2019.pdf.

[27] Vorel A, John F, Hamšíková L. Metodika monitoringu populace bobra evropského v české republice. Příroda. 2006;25:75–94.

[28] Serva D, Biondi M, Mantoni C, Iannella M. Don’t stop it now: functional and structural habitat connectivity assessment suggests further expansion in southern Europe for the Eurasian beaver. Landsc Ecol. 2024;39:25.

[29] Anděra M, Gaisler J. Savci České republiky: popis, rozšíření, ekologie, ochrana = Mammals of the Czech Republic: description, distribution, ecology, and protection. Vydání 1. Praha: Academia; 2012. p. 285.

[30] Nolet BA, Rosell F. Comeback of the beaver Castor fiber: an overview of old and new conservation problems. Biol Conserv. 1998;83(2):165–73. doi: 10.1016/s0006-3207(97)00066-9

[31] Gaywood MJ. Reintroducing the Eurasian beaver *Castor fiber* to Scotland. Mammal Review. 2017;48(1):48–61. doi: 10.1111/mam.12113

[32] Kadlecova G, Voslarova E, Vecerek V. Diurnal raptors at rescue centres in the Czech Republic: Reasons for admission, outcomes, and length of stay. Tubelis DP, editor. PLoS One. 2022;17(12):e0279501. doi: 10.1371/journal.pone.0279501 36584191 PMC9803276

[33] Collins C, Kays R. Causes of mortality in North American populations of large and medium-sized mammals: causes of mortality in mammals. Anim Conserv. 2011;14:474–83.

[34] Borda-de-Água L, Grilo C, Pereira H. Modeling the impact of road mortality on barn owl (*Tyto alba*) populations using age-structured models. Ecol Model. 2014;276:29–37.

[35] Clarke GP, White PCL, Harris S. Effects of roads on badger *Meles meles* populations in south-west England. Biol Conserv. 1998;86:117–24.

[36] Medrano‐Vizcaíno P, Grilo C, Silva Pinto F, Carvalho W, Melinski R, Schultz E. Roadkill patterns in Latin American birds and mammals. Glob Ecol Biogeogr. 2022;31:1756–83.

[37] Vorel A, Korbelová J editors. Průvodce soužití s bobrem. Praha: ČZU v Praze; 2016.

[38] Cunningham JM, Calhoun AJK, Glanz WE. Pond‐breeding amphibian species richness and habitat selection in a beaver‐modified landscape. J Wildl Manag. 2007;71(8):2517–26. doi: 10.2193/2006-510

[39] Williams JE, Neville HM, Haak AL, Colyer WT, Wenger SJ, Bradshaw S. Climate change adaptation and restoration of western trout streams: opportunities and strategies. Fisheries. 2015;40(7):304–17. doi: 10.1080/03632415.2015.1049692

[40] Puttock A, Graham HA, Cunliffe AM, Elliott M, Brazier RE. Eurasian beaver activity increases water storage, attenuates flow and mitigates diffuse pollution from intensively-managed grasslands. Sci Total Environ. 2017;576:430–43. doi: 10.1016/j.scitotenv.2016.10.122 27792958

[41] Härkönen S. Forest damage caused by the Canadian beaver (*Castor canadensis*) in South Savo, Finland. Silva Fenn [Internet]. 1999. [cited 18 November 2024]. Available from: http://www.silvafennica.fi/article/648.

[42] Denoël M, Ficetola G. Conservation of newt guilds in an agricultural landscape of Belgium: the importance of aquatic and terrestrial habitats. Aquat Conserv. 2008;18:714–28.

[43] Nummi P, Hahtola A. The beaver as an ecosystem engineer facilitates teal breeding. Ecography. 2008;31(4):519–24. doi: 10.1111/j.0906-7590.2008.05477.x

[44] Nummi P, Holopainen S. Whole‐community facilitation by beaver: ecosystem engineer increases waterbird diversity. Aquat Conserv. 2014;24:623–33.

[45] Nummi P, Suontakanen E, Holopainen S, Väänänen V. The effect of beaver facilitation on common teal: pairs and broods respond differently at the patch and landscape scales. Ibis. 2019;161:301–9.

[46] Hartman G. Irruptive population development of European beaver (*Castor fiber*) in southwest Sweden. Society for the study and conservation of Mammals, Arnhem. Lutra. 2003;46:103–8.

[47] Kohler F, Andrieu D, Bois E, Cloiseau G, Drelon S, Eggert C. Our neighbor the beaver: anthropomorphism to facilitate environmental mediation in rural France. Hum Ecol. 2023;51:513–28. 10.1007/s10745-023-00406-z PMC10116083 37362024

[48] Mikulka O, Homolka M, Drimaj J, Kamler J. European beaver (*Castor fiber*) in open agricultural landscapes: crop grazing and the potential for economic damage. Eur J Wildl Res. 2020;66:101.

[49] Mikulka O, Homolka M, Drimaj J, Kamler J. Feeding behaviour of Eurasian beavers (*Castor fiber*) along small streams in an agricultural landscape. Acta Univ Agric Silvic Mendelianae Brun. 2022;70:71–82.

[50] Davies JM, Roper TJ, Shepherdson DJ. Seasonal distribution of road kills in the European badger (*Meles meles*). J Zool. 1987;211:525–9.

[51] Moore LJ, Petrovan SO, Bates AJ, Hicks HL, Baker PJ, Perkins SE, et al. Demographic effects of road mortality on mammalian populations: a systematic review. Biol Rev Camb Philos Soc. 2023;98(4):1033–50. doi: 10.1111/brv.12942 36843247

[52] Ferreras P, Aldama J, Beltrán J, Delibes M. Rates and causes of mortality in a fragmented population of Iberian lynx felis pardina temminck, 1824. Biol Conserv. 1992;61:197–202.

[53] Hartman G. Notes on age at dispersal of beaver (*Castor fiber*) in an expanding population. Can J Zool. 1997;75:959–62.

[54] Hartman G. Long‐term population development of a reintroduced Beaver (*Castor fiber*) population in Sweden. Conserv Biol. 1994;8(3):713–7. doi: 10.1046/j.1523-1739.1994.08030713.x

[55] Červený J, Málková P, Bufka L. The current distribution of the beaver (*Castor fiber*) in southwestern Bohemia (Czech Republic). Lynx. 2020;31:13–22.

[56] John F, Baker S, Kostkan V. Habitat selection of an expanding beaver (*Castor fiber*) population in central and upper Morava River basin. Eur J Wildl Res. 2010;56:663–71.

[57] Stocker L. Practical wildlife care. 2nd edition. Somerset: Wiley; 2013.

[58] MacArthur R. Energy metabolism and thermoregulation of beaver (*Castor canadensis*). Can J Zool. 1989;67:651–7.

[59] Goodman G, Girling S, Pizzi R, Meredith A, Rosell F, Campbell-Palmer R. Establishment of a health surveillance program for reintroduction of the Eurasian beaver (*Castor fiber*) into Scotland. J Wildl Dis. 2012;48(4):971–8. doi: 10.7589/2011-06-153 23060498

[60] Saino N. Immune response covaries with corticosterone plasma levels under experimentally stressful conditions in nestling barn swallows (*Hirundo rustica*). Behav Ecol. 2003;14:318–25.

[61] Nitsche KA. The wolf canis lupus as natural predator of beavers *Castor fiber* and *Castor canadensis*. Russ J Theriol. 2016;15:62–7.

[62] Rosell F, Czech A. Responses of foraging Eurasian beavers *Castor fiber* to predator odours. Wildl Biol. 2000;6:13–21.

[63] Campbell-Palmer R, Rosell F. Captive care and welfare considerations for beavers. Zoo Biol. 2015;34(2):101–9. doi: 10.1002/zoo.21200 25653085

[64] Campbell-Palmer R, Del Pozo J, Gottstein B, Girling S, Cracknell J, Schwab G, et al. Echinococcus multilocularis detection in live Eurasian beavers (*Castor fiber*) using a combination of laparoscopy and abdominal ultrasound under field conditions. PLoS One. 2015;10(7):e0130842. doi: 10.1371/journal.pone.0130842 26167927 PMC4500463

[65] Marreros N, Zürcher-Giovannini S, Origgi FC, Djelouadji Z, Wimmershoff J, Pewsner M, et al. Fatal leptospirosis in free-ranging Eurasian beavers (*Castor fiber* L.), Switzerland. Transbound Emerg Dis. 2018;65(5):1297–306. doi: 10.1111/tbed.12879 29673086

[66] Maas M, Glorie J, Dam-Deisz C, de Vries A, Franssen FFJ, Jaarsma RI, et al. Zoonotic pathogens in eurasian beavers (*Castor fiber*) in the Netherlands. J Wildl Dis. 2022;58(2):404–8. doi: 10.7589/JWD-D-21-00097 35245369

[67] Serva D, Biondi M, Iannella M. The eurasian beaver range expansion reveals uneven future trends and possible conservation issues: an European assessment. Biodivers Conserv. 2023;32:1999–2016.

